# Sedentary behaviour levels in adults with an intellectual disability: a systematic review protocol

**DOI:** 10.12688/hrbopenres.13123.2

**Published:** 2021-03-29

**Authors:** Louise Lynch, Mary McCarron, Philip McCallion, Eilish Burke

**Affiliations:** 1 School of Nursing and Midwifery, Trinity College, Dublin, Ireland; 2 School of Social work, College of Public Health, Temple University, Philadelphia, Pennsylvania, USA

**Keywords:** Intellectual disability, sedentary behaviour, adults

## Abstract

**Background:** Sedentary behaviour contributes to non-communicable diseases, which account for almost 71% of world deaths. Of these, cardiovascular disease is one of the largest causes of preventable death. It is not yet fully understood what level of sedentary behaviour is safe. People with an intellectual disability have poorer health than the general population with higher rates of multi-morbidity, obesity and inactivity. There is a paucity of evidence on whether this poorer health is due to sedentary behaviour or physical inactivity. This systematic review will investigate the sedentary behaviour levels of adults with an intellectual disability.

**Method**: The PRISMA-P framework will be applied to achieve high-quality articles. An extensive search will be conducted in Medline, Embase, psycINFO and Cinahl and grey literature sources. All articles will be independently reviewed by two reviewers and a third to resolve disputes. Initially, the articles will be reviewed by title and abstract and then the full article will be reviewed using stringent inclusion criteria. All article data will be summarised in a standardised tabular format. The National Institute of Health’s quality assessment tool will be used to assess article quality. GRADE will be used to assess the quality of the evidence. The primary outcome of interest is the prevalence of sedentary behaviour levels for people with an intellectual disability. The definition of sedentary behaviour to be used for the purposes of this study is: ‘low physical activity as identified by metabolic equivalent (MET) or step levels or as measured by the Rapid Assessment of Physical activity questionnaire (RAPA) or the International Physical Activity questionnaire (IPAQ) or sitting for more than 3 hours per day’.

**Conclusion: **This systematic review will provide a critical insight into the prevalence of sedentary behaviour in adults with an intellectual disability.

## Introduction

### Rationale

According to the World Health Organisation ( WHO, 2013), non-communicable diseases account for almost 71% of world deaths. Non-communicable diseases are non-infectious and chronic but can be prevented. Of these, cardiovascular disease (CVD) is one the largest causes of preventable death worldwide with over 17.9 million dying annually. CVD can manifest as increased blood pressure or elevated blood lipid levels, leading to heart attack or stroke. One of the main contributors to CVD is lack of physical activity ( Forouzanfar *et al*., 2016). Physical activity is any bodily movement which uses skeletal muscles and results in energy expenditure ( WHO, 2019) while a sedentary lifestyle is one which has low levels of physical activity and consequently low levels of energy expenditure. In general, people with intellectual disability (ID) have poorer health than their non-disabled contemporaries ( Emerson *et al*., 2016) and often experience health disparities ( Krahn & Fox, 2014). However, the real state of the science regarding sedentary behaviour and people with ID is not known. Further investigation is essential to understand if sedentary behaviour contributes to these health differences.

It is necessary to understand some of the known contributors to CVD, obesity and physical inactivity, as well as sedentary behaviours because these are all modifiable and inter-related health risks factors.

***Sedentary behaviour.*** Sedentary comes from the Latin word *sedere* which means to sit and can describe a wide range of distinct activities which require low levels of energy expenditure in any setting ( Thorp *et al*., 2011). The first real attempt to define the term ‘sedentary’ was made in 2012 ( Tremblay *et al*., 2017). This was in an effort to avoid confusion by standardising the terms to refer to sedentary or inactive behaviours used in journals. A metabolic equivalent (MET), known as the resting metabolic rate, is an objective measurement scale used to classify activity types and levels. A MET is the amount of oxygen (O _2_) burned at rest and is the equivalent of 3.5ml O _2_ per kg body weight per minute ( Jette *et al*., 1990) or 1kilocalorie per kg of body weight per hour ( Newton *et al*., 2013). Tremblay *et al.* (2017) proposed to define sedentary behaviour as ‘any waking behaviour characterized by an energy expenditure of ≤1.5 METs while in a sitting or reclining posture’ for example watching television or working on a computer. Hence sedentary behaviour constitutes too much sitting or stationary activity as opposed to physical inactivity which is too little exercise or physical movement. Tudor-Locke *et al.* (2013) found a link between reduced steps per day (less than 5,000) and being more sedentary. In addition, sitting for prolonged periods (more than 3 hours per day) has been found to have adverse health effects ( Pinto Pereira *et al*., 2012).

Sedentary behaviour has been linked to adverse health conditions in older adults, increased cardio metabolic risks, increased obesity and mortality in both men and women, as well as increased cancer risk ( de Rezende *et al*., 2014; Patel *et al*., 2010; Same *et al*., 2016; Thorp *et al*., 2011). Self-reported studies have shown that high levels of sedentary behaviour, even if minimum exercise guidelines are met, show increased metabolic risk ( Patel *et al*., 2010). This impact of sedentary behaviour can be mollified by interspersing periods of physical activity throughout the day ( Healy *et al*., 2008).

An ecological model of sedentary behaviour for older adults without an ID, proposed by Owen *et al.* (2011), could be used to assess the sedentary behaviours of people with ID. This model classed sedentary behaviour into four categories:

- Household (e.g. watching TV)
- Leisure time (increased screen-based and sitting activities)
- Transport (driving, sitting on public transport to/from work/activities)
- Occupation (e.g. screen-based computer work).

**Sedentary behaviour and people with ID**

In a systematic review by Melville *et al.* (2017), it was proposed that studies to determine sedentary behaviour in people with ID did not use enough randomly selected samples and sample sizes were too small, meaning that results could not be generalised for the ID population as a whole. Furthermore, insufficient studies have distinguished between sedentary behaviour and inadequate physical activity. Westrop *et al*., 2019 in a systematic review investigating physical activity and sedentary behaviour in adults with an intellectual disability deduced that men were more active than women but that results on sedentary behaviour were inconclusive due to insufficient studies. Consequently, it is not clear what the actual sedentary behaviour of people with ID is.

Older people with an intellectual disability have been shown to have higher rates of multi-morbidity, obesity and inactivity than the general population ( Gawlik *et al*., 2018; McCarron *et al*., 2013). In 2016 approximately 70,000 people, 1.4% of the overall Irish population ( Census, 2016), were shown to have an ID. In an analysis of secondary data, Harris *et al.* (2017) deduced that people with ID were sedentary for over 70% a day. According to Graham & Reid (2000), adults with ID are more susceptible to age-related health risks, where sedentary behaviour could be a contributing factor.

While breaking up time spent doing sedentary activity has been shown to increase daily living activities and physical independence in older adults ( Sardinha *et al*., 2015), there is no similar information on adults with ID. Increased sedentary behaviour has been linked to obesity levels and increased likelihood of multi-morbidity ( Melville *et al*., 2017), but inconsistent evidence exists on links of sedentary behaviour to level of ID ( Oppewal *et al*., 2018). Often studies use proxy measures (e.g. watching TV) to determine sedentary behaviour which may be inaccurate, especially with regards to people with ID, as people with a more severe ID may be less likely to watch TV due to sensory or cognitive impairments ( Owen *et al*., 2011). Level of ID has been shown to be directly related to physical activity but not sedentary behaviour ( Oppewal *et al*., 2018).

Emerging evidence is highlighting the importance of reducing sedentary behaviour for improving cardio-metabolic health and adopting a holistic public health approach to improve activity levels as well as sedentary behaviour ( van der Ploeg & Hillsdon, 2017). 

For the purposes of this systematic review, sedentary behaviour will be defined as:

‘low physical activity as identified by MET or step levels or as measured by the Rapid Assessment of Physical activity questionnaire (RAPA) or the International Physical Activity questionnaire (IPAQ) or sitting for more than 3 hours per day’

***Obesity.*** Globally almost 38% of the world’s population, a greater than 100% increase since 1980, and two-thirds of the American population, are either overweight or obese, with a BMI of greater than 25.0 kg/m ^2^ ( Fryar *et al*., 2012; Ng *et al*., 2014; WHO, 2009). In Ireland, almost 23% of adults are obese with 50% of women and 66% of men being overweight ( Ng *et al*., 2014. This is a huge concern given the proven link between obesity and cancers, higher rates of type II diabetes, CVD and CVD mortality ( Bhaskaran *et al*., 2014; Hossain *et al*., 2007; Ortega *et al*., 2016).

**Obesity and people with ID**

A 2017 review found that the prevalence of overweight and obesity in people with ID varies from 28%–71% and 17%–43%, respectively ( Ranjan *et al*., 2018). The IDS-TILDA study found that overweight and obesity in people with ID increased from 66% in wave2 to 79.7% in wave3 and that 64% of participants considered themselves to be at the right weight ( Burke *et al*., 2017). According to a US based longitudinal study on people with ID women appear to be at a greater risk of developing morbid obesity while men were more likely to be overweight ( Hsieh *et al*., 2014)).

According to Fock & Khoo (2013), excessive calorific intake and increased sedentary behaviour are the main contributors to increased obesity levels but obesity levels may be ameliorated by a combination of healthy eating, a reduction in sedentary behaviour and an increase in physical activity

***Physical inactivity.*** Physical inactivity is classified as not meeting the minimum activity requirements. According to the American College of Sports Medicine, moderate-intensity aerobic physical activity (PA) of between 150 and 250 minutes per week is the minimum necessary for health and weight management in adults ( Donnelly *et al*., 2009; Health Service Executive, 2009; US Department of Health, 2018). Insufficient PA or physical inactivity contributes to adverse health issues like obesity, CVD and cancer as well as increased mortality ( Lee *et al*., 2012). According to the World Health Organisation ( WHO, 2009), physical inactivity is the fourth leading risk factor for all-cause mortality, with over three million deaths annually. Of concern is that Ireland is one of the least active countries in Europe ( Loyen *et al*., 2016).

**Physical inactivity and people with ID**

For People with an ID, the amount of moderate PA done, and the number of hours spent watching TV was found to be significantly associated with obesity level ( Hsieh *et al*., 2014). A 2016 Australian based study found that over 66% of participants did not meet minimum exercise guidelines ( Koritsas & Iacono, 2016), while another study found that 77% of participants did not meet the minimum exercise recommendations ( Barnes *et al*., 2013). It must be noted that physical impairments leading to the use of walking aids or wheelchairs may inhibit physical activity for some people with an ID ( Ranjan *et al*., 2018).

Hence sedentary behaviour and physical inactivity are different and should be addressed separately with distinct guidelines for each. While specific recommendations for movement and physical activity levels in adults have been long established, corresponding recommendations for sedentary behaviour have not. The recommendations from emerging evidence are to minimise the amount of time being sedentary, but no specifics have yet been established for the general population or people with ID ( WHO, 2020).

### Developing the question

A focused and well-defined question avoids bias in literature searches, ensures clarity and therefore ensures the identification of the concepts for the focused search. PICO, which is used for quantitative studies is being used to define the question as follows ( Schardt *et al*., 2007):

- P [Population or problem]: Adults aged 18+ with an Intellectual Disability
- I [Intervention or exposure]: Sedentary behaviour level (SB in line with the definition of SB defined for this review
- C [Comparison]: Individuals with all levels of ID living in residential, community group homes, with family or independently
- O [Outcome]: Prevalence of Sedentary behaviour

The research question to be addressed by this systematic review protocol is

*‘What are the sedentary behaviour levels of older Adults with an Intellectual Disability?’.*

## Methods

PRISMA-P, for the reporting and development of systematic review protocols is used as the guide in the writing of this protocol ( Shamseer *et al*., 2015). The completed PRISMA-P checklist for this protocol is available as extended data ( Lynch, 2020).

### Eligibility criteria

The criteria for inclusion in the review are as follows:

- Population: adults aged 18+ with an Intellectual Disability
- Language: English
- Study type: All types of studies including primary studies, peer reviewed, grey literature
- Study design: Randomised controlled trials, cohort, cross-sectional
- Content: Must reference sedentary behaviours of adults with ID to be eligible for inclusion
- Timeframe: no restriction on timeframes up to March 2020.

The criteria for exclusion in the review are as follows:

- Population: Children with or without an ID and Adults without ID
- Language: Articles that are not available in English
- Study design: Any type of reviews
- Conference proceedings and published conference abstracts only

### Information sources

***Databases***

The following four databases will be used to perform the search:

Medline
Embase
psycINFO
Cinahl

In addition, the following sources will be explored for grey literature sources:

The CORDIS library
Grey Literature Database from the Canadian Evaluation Society
The U.S. Department of Housing and Urban Development (HUD) User database
National Technical Information Service (NTIS)
Open Grey
Social Care Online
Social Science Research Network (SSRN) eLibrary
RIAN
Google Scholar
Proquest (Dissertations and Theses)

### Search strategy

**Figure 1.**
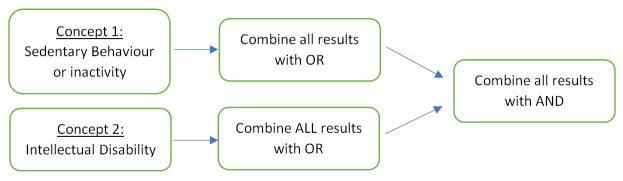
Search strategy.

The search strategy was refined into two concepts following the application of PICO. Concept 1 is ‘Sedentary behaviour or inactivity’ and Concept 2 is ‘Intellectual Disability’. Each of the two concepts will be searched using MESH terms and keywords and then combined using OR. Then the total results of each concept will be combined using AND (See Figure 1). This search will be repeated for each of the four databases. The resulting article list will be the complete combined database search results. This list will be screened for inclusion.

***Search string.*** An example of the search string used for the Medline database is shown in Table 1.

**Table 1.** Medline search string.

Concept	Index	Keywords
**Concept 1:** Sedentary behaviour & physical inactivity	(MH "Sedentary Behavior")	sedentary lifestyle* OR sedentary behavior* OR sedentary behaviour* OR physical* inactiv* OR inactive lifestyle
**Concept 2:** Intellectual disability or learning disability	(MH "Intellectual Disability+") OR (MH "Learning Disabilities+")	((intellectual AND disabilit* OR 'mental retardation'/exp OR 'mental retardation' OR (mental AND ('retardation'/exp OR retardation)) OR 'learning'/ exp OR learning) AND disabilit* OR developmental) AND disabilit* OR 'learning disabilities'/exp OR 'learning disabilities' OR (('learning'/exp OR learning) AND disabilities)

***Screening process.*** All identified articles from each database that is searched, as well as all grey literature sources, will be combined and duplicates removed. Endnote software will be used to store all the identified articles. The articles will be stored in folders which are named after the search process used. Using the inclusion criteria as detailed above, all articles will initially be screened by title and then by abstract. The remaining full text articles will be retrieved and read thoroughly. Those that do not meet the inclusion criteria will be omitted. The remaining articles will then be quality assessed using two separate assessors with a third person as an adjudicator should any discrepancies arise.

***Quality assessment and risk of bias.*** The remaining articles will be assessed using two validated quality assessment tools from the National Institute of Health ( National Institute Health, 2020), the first for observational cohort and cross-sectional studies and the second for randomised controlled trials (RCTs). The tools used are available as extended data ( Lynch, 2020).

These tools are used to critically assess the internal validity of each article and identify any issues or sources of potential bias. According to Cochrane, effectively evaluating the quality of a study is done by looking at its design, methodology, results, analysis and reporting, and how they relate to the original research question ( Higgins *et al*., 2011).

There are different types of study quality assessment tools for the different study types. For Controlled Intervention Studies and Observational Cohort and Cross-sectional studies, 14 criteria are used to evaluate the study quality, while for Case-Control studies 12 criteria are used. 11 criteria are used to determine the study quality of RCTs. This means that a maximum quality score of 11, 12 or 14 can be achieved depending on the study type. This quality score will be used to determine if the study should be included in the review. Quality scores are divided into 3 main categories: Good, Fair or Poor. See Table 2 for details.

**Table 2.** Quality assessment Scoring System.

Quality Rating	Observational Cohort & Cross-Sectional Studies	Case- Control Studies	RCTs	Action
Good	9 - 12	10 - 14	7 - 11	Data extraction
Fair	6 - 8	7 - 9	4 - 6	2 reviewers to discuss. Adjudicate with 3rd reviewer if required.
Poor	<=5	<= 6	<= 4	2 reviewers to discuss. Reject
Other	CD, NR, NA *			

* CD = Cannot determine, NR = Not reported, NA = Not applicable

Any studies that are excluded will be tracked with reasons for rejection.

### Quality scoring

Scores are attributed to distinct parts of the study design for example type of study, design and blinding, where a ‘yes’ answer gives a score of ‘1’, a ‘no’ answer a score of ‘0’ and could potentially highlight an issue with the article. See Table 3.

**Table 3.** Study assessment scoring.

Answer	Score
Yes	1
No	0
Cannot determine/not reported/not applicable	0

### Ethics

This research project is part of the IDS-TILDA project. Full ethical approval for IDS-TILDA has been granted by the Trinity College Dublin Faculty of Health Sciences Research Ethics Committee.

## Study records

### Data management

All search records will be kept in an excel spreadsheet detailing the database, type of search (keyword or MESH terms) and the resulting search numbers. The articles will be stored in Endnote. Each stage of the search and review will be recorded in excel. For each stage of the search process, articles will be stored in an appropriately named folder in EndNote X9 for windows.

### Selection process

The selection process of studies for inclusion, which are identified by the search strategy, will be done by two independent review authors [LL and EB]. The initial screening will be done by title and abstract. If eligibility is inconclusive from the title and abstract, the full text of the article will be assessed. Any articles that do not match the inclusion criteria will be excluded. Any differences on article inclusion between the two authors will be resolved by discussions with the third review author [MMc]. Finally, the full-text article of all potential articles that could be included in the review will be independently assessed by the authors for inclusion as above.

### Data collection process

An excel spreadsheet will act as the data extraction tool. This will be used to summarise all the shortlisted studies. The categories to be captured are as in Table 4 ( McKenzie *et al*., 2019).

**Table 4.** Article Data Collection Categories.

**Author, title, year**
- study focus
- study type
- Intervention type
- country
- duration
- dates
- numbers
**Participants**
- number
- mean age
- gender (%)
- level of ID
- living circumstances
- employment type
**Assessment**
- type
- intervention
- Assessment type
- measurement device
- outcome/data
- Statistical results
**Findings**
**Summary**
**Comment**

### Data items

The PICO framework will be used to define what data will be sought from variables as follows:

- P: Adults with an Intellectual Disability
  - Age, gender, living circumstance, country, number in study, level of ID
- I: Sedentary behaviour
  - Level, types of behaviour, quantify change
- C: Level of sedentary behaviour or physical inactivity
  - Level, intensity, types of activity/sedentary behaviour, type of employment
- O: Prevalence of sedentary behaviour

### Outcomes and prioritisation

The outcomes of this investigation into sedentary behaviour will determine the sedentary behaviour levels of older adults with an intellectual disability.

***Primary outcome***

- Sedentary behaviour levels

### Data synthesis

If quantitative studies are homogenous in nature a meta-analysis may be performed and a forest plot produced to summarise results. A narrative synthesis will be used to summarise all the study article data and relevant information. For qualitative studies, a thematic analysis of the semantic and latent topics of the articles using a 6-step process (see Table 5), will guide the derivation of a framework for the analysis of the outcome data ( Braun & Clarke, 2006).

**Table 5.** 6-step thematic analysis process.

Step number	Process	Explanation
**1**	Data familiarisation	Complete data immersion
**2**	Generate initial codes	Topics, patterns of data
**3**	Search for themes	Broader theme identification
**4**	Review of themes	Theme refinement
**5**	Define and name themes	Categorise. Include sub-themes if required
**6**	Produce report	Complete write-up

Statistical comparisons of article data will be reviewed on a case-by-case basis.

### Confidence in cumulative evidence

The GRADE (Grading of Recommendations Assessment, Development and Evaluation) approach will be used to assess the strength of the body of evidence of the review. In line with the Cochrane methodology, each outcome will be ranked according to whether the quality is high, moderate, low or very low. The GRADE framework will be used to assess each outcome in the following areas: risk of bias, consistency of effect, imprecision, indirectness and publication bias ( Schünemann *et al*., 2019).

## Dissemination of information

The dissemination plan will be to present at conferences for example the THEconf March 2021, Irish Gerontology Society PhD event and other ID or physical activity events or conferences as well as publishing in journals.

## Study status

Searches are currently in progress.

## Conclusion

This systematic review of the sedentary behaviour levels of older adults with an intellectual disability will provide a critical insight into the sedentary behaviours of this population group.

## Data availability

### Underlying data

No data are associated with this article

### Extended data

Harvard Dataverse: Replication Data for: Sedentary behaviour levels in adults with an intellectual disability: a systematic review protocol. https://doi.org/10.7910/DVN/TPS2HU ( Lynch, 2020)

This protocol contains the following extended data:

- PRISMA-P checklist
- Study Quality Assessment Tools for observational, cohort, cross-sectional and randomised controlled trials

### Reporting guidelines

Harvard dataverse: PRISMA-P checklist for ‘Sedentary behaviour levels in adults with an intellectual disability: a systematic review protocol’ https://doi.org/10.7910/DVN/TPS2HU ( Lynch, 2020)
