# Supplementary material for: Sedentary behaviour levels in adults with an intellectual disability: a systematic review protocol
Source: HRB Open Res. 2021 Mar 29;3:57. Originally published 2020 Aug 26. [Version 2] doi: 10.12688/hrbopenres.13123.2 (PMC8063547; doi:10.12688/hrbopenres.13123.2)
Supplement: Peer review history

# Peer review: Reviewer response for version 2

**DOI:** 10.21956/hrbopenres.14429.r29218

**Published:** 2021-04-13

**Authors:** Thessa Hilgenkamp

**Affiliations:** 1 Department of Physical Therapy, University of Nevada, Las Vegas, NV, USA

In this revision, the authors included the specific quality assessment tools they intend to use, which greatly improves the replicability of the results. I have no further comments.

Is the study design appropriate for the research question?

Yes

Is the rationale for, and objectives of, the study clearly described?

Yes

Are sufficient details of the methods provided to allow replication by others?

No

Are the datasets clearly presented in a useable and accessible format?

Not applicable

Reviewer Expertise:

Physical activity, fitness, intellectual disabilities, Down syndrome, exercise physiology.

I confirm that I have read this submission and believe that I have an appropriate level of expertise to confirm that it is of an acceptable scientific standard.

# Peer review: Reviewer response for version 2

**DOI:** 10.21956/hrbopenres.14429.r29219

**Published:** 2021-04-12

**Authors:** Catherine Ruth Hankey

**Affiliations:** 1 College of Medical Veterinary and Life Sciences, Institute of Mental Health and Wellbeing, University of Glasgow, Glasgow, UK

Having looked over the revised article I am very pleased with the revisions that have been made by the authors of this review. I am happy to alter the article status from approved with reservations to approved.

Is the study design appropriate for the research question?

Yes

Is the rationale for, and objectives of, the study clearly described?

Yes

Are sufficient details of the methods provided to allow replication by others?

Yes

Are the datasets clearly presented in a useable and accessible format?

Not applicable

Reviewer Expertise:

Research experience in the field of lifestyle in those adults with ID.

I confirm that I have read this submission and believe that I have an appropriate level of expertise to confirm that it is of an acceptable scientific standard.

# Peer review: Reviewer response for version 1

**DOI:** 10.21956/hrbopenres.14233.r28947

**Published:** 2021-03-05

**Authors:** Thessa Hilgenkamp

**Affiliations:** 1 Department of Physical Therapy, University of Nevada, Las Vegas, NV, USA

This protocol for a systematic review addresses a relevant and important topic for individuals with intellectual disabilities. Due to the current interest in this topic, this update of the previous 2017 systematic review on this topic is likely to include some newer studies published in the last 3 years. The search strategy is very inclusive, and the inclusion and exclusion criteria are clearly described. However, the quality assessment and risk of bias is not clearly described, which is a key element to a systematic review protocol. The tool included as extended data is a tool to evaluate the quality of a systematic review as a whole, not to assess the papers included in a systematic review. The authors then mention there are different types of study quality assessment tools for the different study types, and a number of criteria used to evaluate those study types, but it is not clear whether this is based on an existing and validated tool, or a self-designed list of criteria, nor is it clear which criteria these lists consist of. Referencing this tool or making this tool/list available as extended data would greatly improve the replicability of the methods.

Is the study design appropriate for the research question?

Yes

Is the rationale for, and objectives of, the study clearly described?

Yes

Are sufficient details of the methods provided to allow replication by others?

No

Are the datasets clearly presented in a useable and accessible format?

Not applicable

Reviewer Expertise:

Physical activity, fitness, intellectual disabilities, Down syndrome, exercise physiology.

I confirm that I have read this submission and believe that I have an appropriate level of expertise to confirm that it is of an acceptable scientific standard, however I have significant reservations, as outlined above.

# Response

**Published:** 2021-03-13

**Authors:** Louise Lynch

**Affiliations:** Trinity College Dublin, Ireland

Thank you for taking the time to review my protocol for a Systematic review of sedentary behaviour in adults with an intellectual disability.

 The correct National Institute Health (NIH) tools for observational, cohort, cross-sectional and randomised controlled trials have now been included in the extended data and the tool for assessing systematic reviews removed. In addition a reference which shows all quality assessment tools by the NIH is included. Furthermore the criteria for RCTS has been added to the table. Wording has been changed to highlight that the tools being used for quality assessment are validated tools.

# Peer review: Reviewer response for version 1

**DOI:** 10.21956/hrbopenres.14233.r28181

**Published:** 2021-01-07

**Authors:** Catherine Ruth Hankey

**Affiliations:** 1 College of Medical Veterinary and Life Sciences, Institute of Mental Health and Wellbeing, University of Glasgow, Glasgow, UK

This is an interesting topic - and the methods that have been proposed are robust. However, my concerns are that the topic of physical activity in those adults with ID has already been recently examined by Westrop SC in 2019 ^1^. This work, a systematic review, I would expect to have examined much the same area that is proposed in this proposed new systematic review. The Westrop publication has not been included in this protocol and should be.

 Having looked at the published research - it seems that there are no systematic reviews in those children and young adults with ID with regard to their physical activity.

 I think that the application is well written and clear despite being very similar to the Westrop work which was published last year. The field of ID is a small one and I imagine that there are not sufficient new works to lead to any differing conclusions than those of Westrop. However, I understand that the HRB are keen to support reanalysis of literature and therefore indexing of this protocol may be justified. 

 Thank you for inviting me to review this work.

Is the study design appropriate for the research question?

Yes

Is the rationale for, and objectives of, the study clearly described?

Yes

Are sufficient details of the methods provided to allow replication by others?

Yes

Are the datasets clearly presented in a useable and accessible format?

Not applicable

Reviewer Expertise:

Research experience in the field of lifestyle in those adults with ID.

I confirm that I have read this submission and believe that I have an appropriate level of expertise to confirm that it is of an acceptable scientific standard, however I have significant reservations, as outlined above.

## References

[1] : Gender differences in physical activity and sedentary behaviour in adults with intellectual disabilities: A systematic review and meta-analysis. *J Appl Res Intellect Disabil*.2019;32(6) : 10.1111/jar.12648 1359-1374 10.1111/jar.12648 31373127

# Response

**Published:** 2021-03-13

**Authors:** Louise Lynch

**Affiliations:** Trinity College Dublin, Ireland

Thank you for taking the time to review my protocol for a systematic review on the sedentary behaviour levels in adults with an intellectual disability.

 The Westrop Systematic review has now been included in the introduction to this protocol. The Westrop review focussed on the gender differences of physical activity and sedentary behaviour while this systematic review will look at the overall prevalence of sedentary behaviour. In addition this systematic review will endeavour to do comparisons by age, level of ID and residence if studies are available. This review will include a comprehensive grey literature search as well as any updated articles from the standard database search. Hence it will add to the body of evidence on sedentary behaviour which is sparse for people with ID.
